# Biodegradable Surgical Staple Composed of Magnesium Alloy

**DOI:** 10.1038/s41598-019-51123-x

**Published:** 2019-10-11

**Authors:** Hizuru Amano, Kotaro Hanada, Akinari Hinoki, Takahisa Tainaka, Chiyoe Shirota, Wataru Sumida, Kazuki Yokota, Naruhiko Murase, Kazuo Oshima, Kosuke Chiba, Yujiro Tanaka, Hiroo Uchida

**Affiliations:** 1 Department of Pediatric Surgery, Graduate School of Medicine, The University of Tokyo, Tokyo, 113-8655 Japan; 2 Advanced Manufacturing Research Institute, National Institute of Advanced Industrial Science and Technology (AIST), Ibaraki, 305-8564 Japan; 3 Department of Pediatric Surgery, Nagoya University Graduate School of Medicine, Nagoya, 466-8550 Japan

**Keywords:** Gastroenterology, Materials for devices

## Abstract

Currently, surgical staples are composed of non–biodegradable titanium (Ti) that can cause allergic reactions and interfere with imaging. This paper proposes a novel biodegradable magnesium (Mg) alloy staple and discusses analyses conducted to evaluate its safety and feasibility. Specifically, finite element analysis revealed that the proposed staple has a suitable stress distribution while stapling and maintaining closure. Further, an immersion test using artificial intestinal juice produced satisfactory biodegradable behavior, mechanical durability, and biocompatibility *in vitro*. Hydrogen resulting from rapid corrosion of Mg was observed in small quantities only in the first week of immersion, and most staples maintained their shapes until at least the fourth week. Further, the tensile force was maintained for more than a week and was reduced to approximately one-half by the fourth week. In addition, the Mg concentration of the intestinal artificial juice was at a low cytotoxic level. In porcine intestinal anastomoses, the Mg alloy staples caused neither technical failure nor such complications as anastomotic leakage, hematoma, or adhesion. No necrosis or serious inflammation reaction was histopathologically recognized. Thus, the proposed Mg alloy staple offers a promising alternative to Ti alloy staples.

## Introduction

Intestinal anastomosis using staples is widely practiced in surgery nowadays^1–4^. Staples used for this procedure are primarily composed of non–biodegradable titanium (Ti) alloy. Although Ti alloy is strong enough for anastomosis, it resides permanently in the human body. As a result, artifacts owing to its high X–ray absorption coefficient can interfere with the examination of patients using computed tomography and lead to misdiagnoses in the area around the staples for the rest of their lives. Moreover, some studies have reported the adverse effects of Ti alloy staples, including allergic and foreign–body reactions and adhesion^5–8^. To overcome these disadvantages, biodegradable surgical staples are desirable.

We thus focus on magnesium (Mg) because it is biodegradable^9,10^, and has excellent biocompatibility^11^. Mg-based medical devices have already been clinically used as vascular stents and orthopedic screws^12–15^. There are also research reports of Mg-based clips being used as surgical devices^16–18^, but the number of studies on the application of Mg-based materials as staples are limited^19–21^, mainly owing to the inadequate ductility of those materials^22^. It has also been reported that Mg staples can easily fracture or degrade at the corners of the B-shape after stapling^19^ because of their vulnerability to stress corrosion^23–26^. Another concern is the production of hydrogen gas caused by the rapid corrosion of Mg in physiological environments^27^.

To address the above challenges, we first redesigned the optimal staple shape to a more rounded form without acute bending points to reduce stress concentrations introduced by stapling and anastomosing^28^. We also developed a novel Mg alloy (Mg–2.5 wt.% Nd–1 wt.% Y, FAsorbMg™) with sufficiently high ductility to be finely processed to form the shape of a staple and stapled without fracturing. It has mechanical properties and biodegradability similar to such Mg alloys as MgYREZr and WE43, which have exhibited satisfactory biocompatibility and clinical feasibility^10,12–15^.

This study investigated the safety and feasibility of a surgical staple using this Mg alloy in three steps and compared it with commercial Ti alloy staples. First, a computer simulation was used to evaluate the deformation behavior of our proposed staple design. Next, we evaluated their biocompatibility, degradation behavior, and mechanical durability using artificial intestinal juice *in vitro*. Finally, the safety and feasibility of the proposed staples were investigated *in vivo* through intestinal anastomoses in porcine models.

## Results

### Deformation behavior of staples

**Figure 1:**
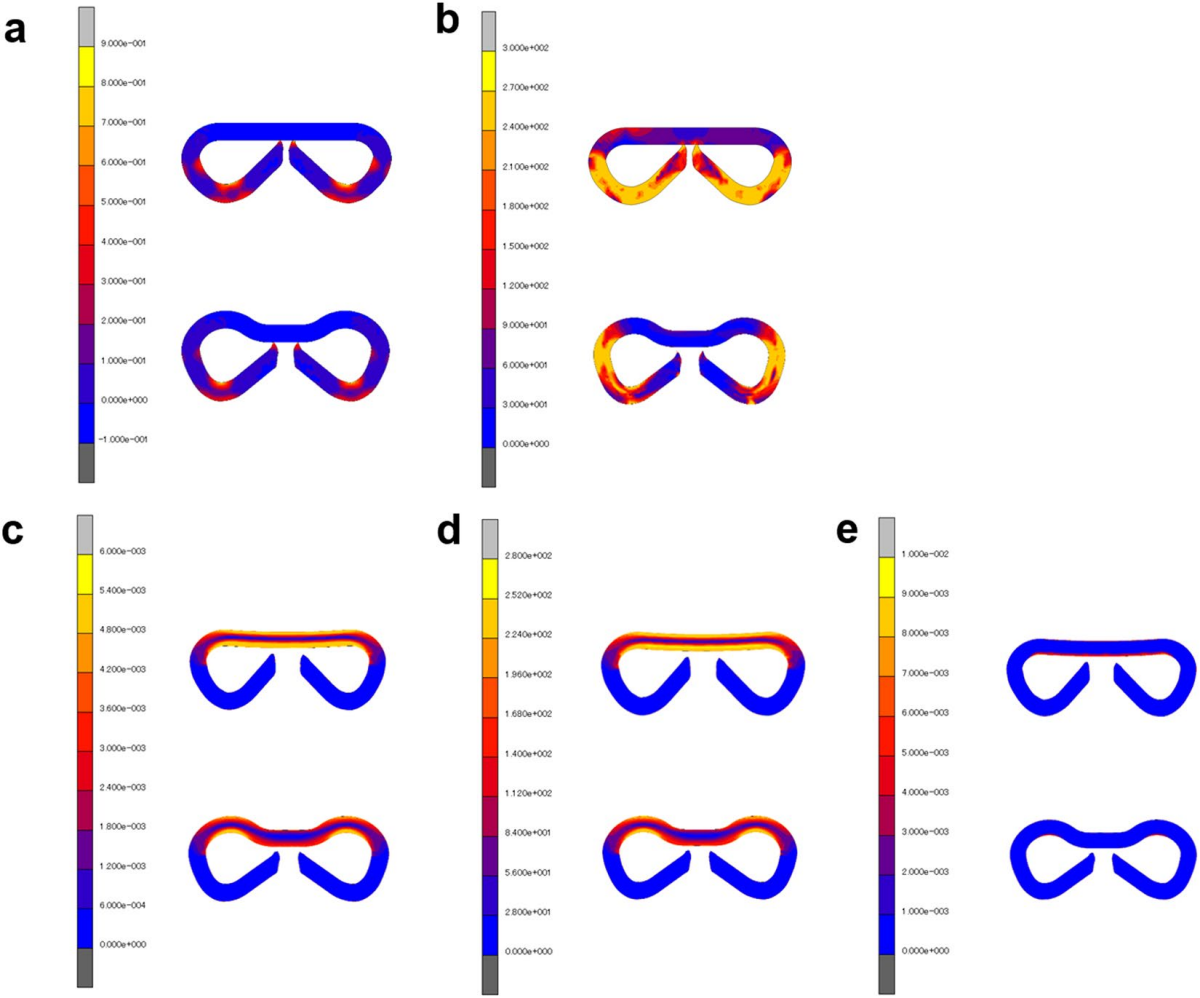
Finite element analysis of deformation behaviors for the conventional staple design (upper) and our staple design (lower). (**a**) Equivalent plastic strain distribution in stapling. (**b**) Equivalent stress distribution in stapling. (**c**) Equivalent elastic strain distribution of the anastomosing staple. (**d**) Equivalent stress distribution of the anastomosing staple. (**e**) Equivalent plastic strain distribution of the anastomosing staple.

Finite element analysis (FEA) revealed that when the B-shape was formed in the stapling, the new staple design relieved the plastic strain concentration compared with the conventional staple design (Fig. 1a). It also revealed that the conventional staple design had stress concentration in all legs while the new design controlled it in a part of the legs (Fig. 1b).

With regard to B-shape retention in intestinal anastomosis, the new staple design can relieve both the strain and stress concentrations at the joint of the legs compared with the conventional staple design (Fig. 1c–e).

### *In vitro* experiments

#### Immersion test

**Figure 2:**
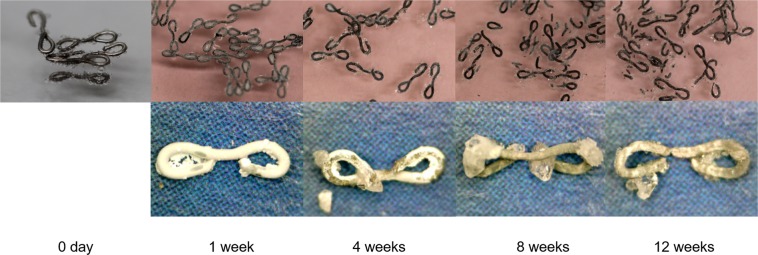
Immersion test using artificial intestinal juice. Macroscopic view of the developed Mg alloy staples at zero, one, four, eight, and 12 weeks after immersion (above) and Mg alloy staples removed after immersion (lower). The surfaces of the staples were covered with a layer of white corrosion product.

Degradation behavior: Macroscopic observation of the Mg alloy staples during the immersion test is shown in Fig. 2. Fine bubbles appeared on the staple surface immediately after immersion but receded shortly thereafter. The products of corrosion covered all staples after four days of immersion. Although the immersed staples degraded gradually, most of them maintained their shapes more than four weeks after immersion.

The concentrations of Mg ions and other elemental components in the artificial intestinal juice are shown in Table 1. The eluted amount of Mg decreased with the period of immersion, and its amount halved after eight weeks of immersion. The concentrations of the eluting elemental components were considerably smaller than that of Mg and were far below the bioactive level.

**Table 1:** Concentrations of elements dissolved from the Mg alloy staples in the immersion test using artificial intestinal juice.

Element	Mg (μg/mL/week)	Y (μg/mL/week)	Nd (μg/mL/week)
1^st^ week	115.667 ± 10.599	<LOD	<LOD
4^th^ week	84.567 ± 4.632	<LOD	<LOD
8^th^ week	50.300 ± 2.651	0.003 ± 0.001	0.005 ± 0.000
12^th^ week	45.433 ± 0.651	<LOD	0.004 ± 0.001

Values are expressed as mean ± standard deviation. <LOD: Below the limit of detection (LOD: Y, Nd; 0.002 µg/mL).

**Figure 3:**
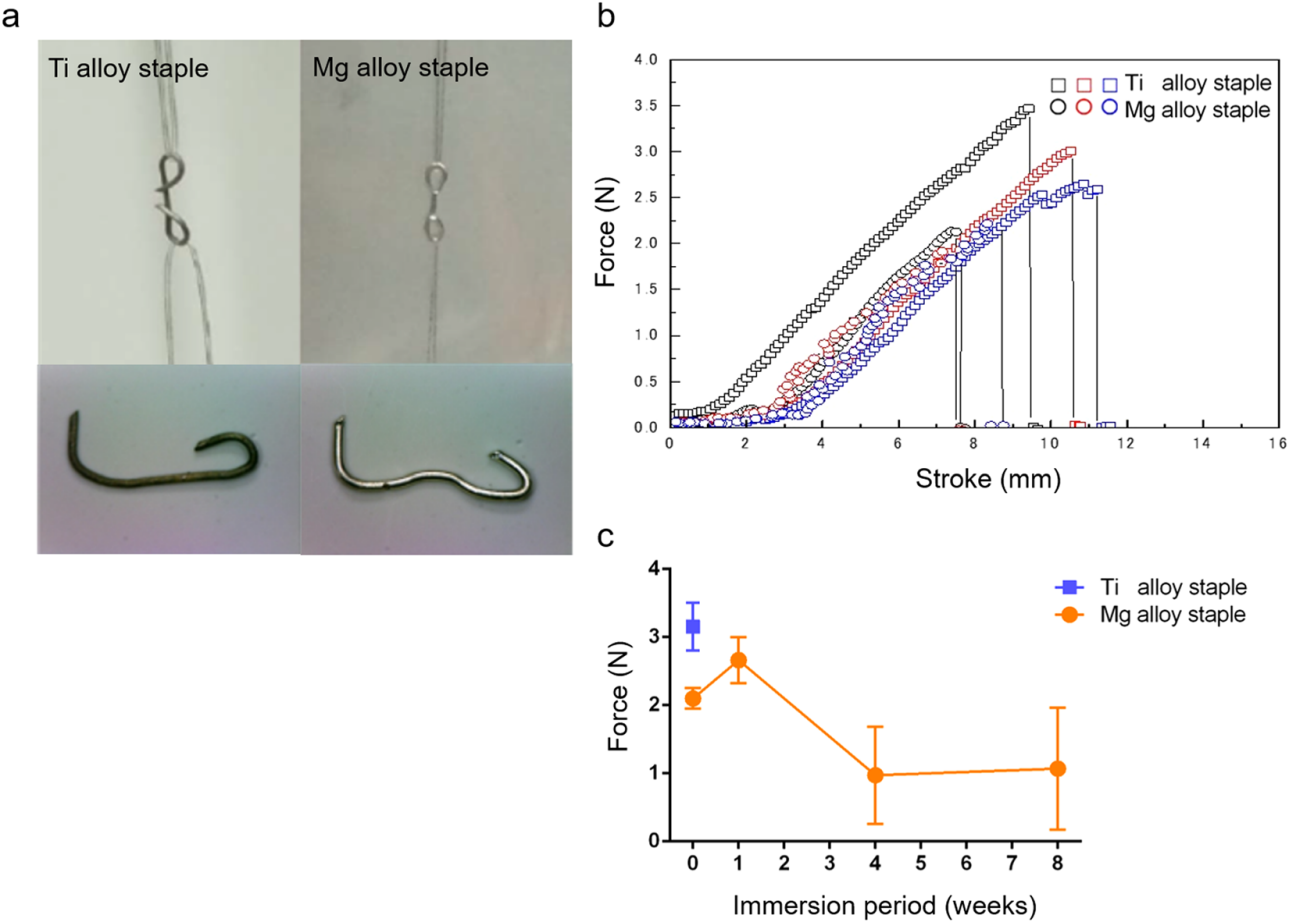
Tensile test. (**a**) Fluorocarbon wires were hooked to each loop of the closing B-shaped staples and loaded in tension until the staple was fractured. (**b**) Tension curve of staples before immersion. (**c**) Results of tensile testing of staples immersed in artificial intestinal juice (bars = mean value with standard deviation).

Mechanical properties: Prior to immersion, the mean tensile force of the Mg alloy staples was 2.10 ± 0.12 N, compared with that of Ti alloy staples of 3.15 ± 0.32 N (Fig. 3a,b). The force of the Mg alloy staples remained at 2.66 ± 0.28 N in the first week of immersion before decreasing to 0.97 ± 0.58 N by the fourth week, followed by 1.07 ± 0.73 N in the eighth week of immersion (Fig. 3c). Mg alloy staples immersed for 12 weeks were not suitable for the tensile test because they were very fragile and shattered easily.

#### Cytotoxicity test

The results of cytotoxicity test are as shown in Table 2. The diluted test solutions suppressed the formation of cell colonies in a concentration-dependent manner. Although no colony was recognizable in 100% extracts, the colony formation rate increased to 73.1% in 50% of the extracts and exceeded 90% in more than 25% of the extracts.

**Table 2:** Colony formation rates of extracts from the Mg alloy staples in the cytotoxicity test.

Concentration of extract (%)	Mg concentration (µg/ml)	Mean colony count	Colony formation rate (%)
0	21.9	43.5	100.0
3.13	50	41.5	95.4
6.25	100	39.8	91.5
12.5	199	41.5	95.4
25	398	42.5	97.7
50	795	31.8	73.1
100	1,590	0.0	0.0

### Experiments on animals

None of the two types of staples caused any technical failure during anastomotic procedures, and all pigs survived the predefined duration. All anastomotic sites of the small intestines healed well, and the Mg alloy staples maintained their shapes without fracture until four weeks after surgery. Any complications, including anastomotic leakage, hematoma, or adhesion, were unrecognizable when the pigs were humanely killed.

**Figure 4:**
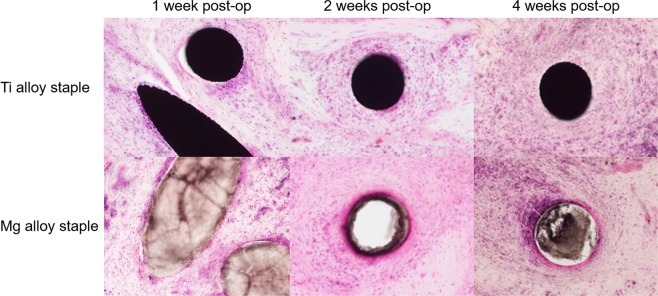
Histopathological images of porcine intestinal tissues surrounding Ti alloy staples and the developed Mg alloy staples (hematoxylin–eosin staining, magnification = x200). Inflammatory cell infiltration around Ti alloy staples was more noticeable than around the developed Mg alloy staples at any point in time.

No necrosis or serious inflammation reaction was histopathologically recognized in the intestinal tissues around the Mg alloy staples. Moreover, the infiltration of inflammatory cells around the staples was less apparent in the Mg group than the Ti group (Fig. 4).

## Discussion

We developed a staple using our original Mg alloy and investigated its clinical potential in simulative, *in vitro* and *in vivo* experiments. Biodegradable Mg alloy-based staples have attracted considerable research attention because of their good biocompatibility^11^ and fewer artifacts in computed tomography^18,29^. However, challenges persist, including insufficient durability, adverse effects associated with biodegradation behavior, and the generation of hydrogen gas in the biodegradation process. Although there are research reports of Mg-based clips^16–18^, the physical properties required for staples differ from those for clips. The diameter of a staple is smaller than that of a clip. Moreover, staples must penetrate the intestine without fracturing and deform into a B-shape (with two bending points).

To maintain sufficient durability for the required period, we employed our original Mg alloy. In addition to its sufficient ductility for staple fabrication, the alloy has excellent yield strength and ultimate tensile strength in comparison with Mg alloys, including high-purity Mg, which has been used to develop surgical staples, as shown in Table 3^12,20,30–32^. Moreover, we designed the shape of a staple to a more rounded form without acute bending points to prevent it from fracturing or degrading at the bending points because Mg is vulnerable to stress corrosion. In this study, the FEA showed that new staple design suitably relieves stress and strain concentration and, consequently, fracture during stapling was reduced and the anastomosing effect was maintained.

**Table 3:** Mechanical properties of Mg alloy, Ti alloy, and colorectal tissue^12,20,30–32^.

Materials	Yield strength (MPa)	Ultimate tensile strength (MPa)	Elongation (%)
FAsorbMg (Mg-2.5%Nd-1%Y)	260 ± 0	290 ± 1	22 ± 1
High-purity Mg	147 ± 8	196 ± 5	14.6 ± 5
WE43	170	220	2
MgYREZr	>250	>275	>10
Ti6Al4V	760–880	830–1,025	12
Colorectal tissue	—	0.87	62.81

Values are expressed as mean ± standard deviation.

The proposed Mg alloy staples maintained their shapes without fracture for more than four weeks and tensile force for at least one week in the artificial intestinal juice, which indicates that they can provide sufficient anastomosing effect for wounds to heal. Mechanical tension in the anastomotic equipment does not necessarily last for months, as shown in Coated VICRYL^®^ (Ethicon Inc., Somerville, New Jersey, USA)—a widely used braided, coated, polyglactin, the strength of which dropped by approximately 25% within two weeks and 75% within four weeks of implantation in rats, according to ref.^33^. We also confirmed the durability of Mg alloy staples by porcine intestinal anastomoses. Intestinal anastomoses stapled using Mg alloy staples were successfully performed, and all the pigs used survived without complications, including anastomotic leakage. Therefore, the proposed Mg alloy staples can provide sufficient anastomosing effect for adequate periods as a device for wound anastomosis.

With respect to the impact of elution, we measured the concentration of the eluted substances in artificial intestinal juice and compared it with the results of the cytotoxicity test. As the colony formation rate lower than 70% is considered cytotoxic according to the International Organization for Standardization (ISO) 10993–5:2009^34^, Mg concentrations of more than 800 μg/mL were considered to invoke a cytotoxic effect. However, the Mg concentration in the immersion test assuming clinical settings was far below 800 μg/mL—the threshold of the cytotoxic effect. The concentrations of the released alloy components other than Mg were much smaller than that of Mg and far below the bioactive level. Moreover, in the experiment involving porcine intestinal anastomoses, no histopathological necrosis or severe inflammatory reaction in the surrounding tissues of Mg alloy staples was observed. Therefore, the proposed Mg alloy staples can be considered biologically safe.

The evolution of hydrogen gas was also assessed in this study. As Mg degrades rapidly in a physiological environment, hydrogen gas can be produced in excess^27^. However, the slow degradation rate of our Mg alloy staples, which were improved with the addition of rare earth elements and the application of grain structure refinement in the fabrication processing, reduced the volume of the gas produced. In the immersion test using artificial intestinal juice, the generation of small amounts of gas was observed only in the first week of immersion. Moreover, in porcine intestinal anastomoses, there were no postoperative complication, including anastomotic leakage or abscess, caused by hydrogen evolution.

This study has a few limitations. First, the impact of eluting Mg in a physiological environment was not fully evaluated mainly because a cytotoxicity test for the biodegradable device has not been developed, and we used an extract with an unreasonably high concentration of Mg in clinical use for the cytotoxicity test. Consequently, the eluting Mg concentration of the 100% extract showed toxic levels in the cytotoxicity test according to ISO 10993–5:2009^34^. As mentioned above, the proposed Mg alloy staple is in the non-toxic level, considering the eluting Mg concentration of Mg alloy staples in clinical use from the immersion test. Moreover, we employed artificial intestinal juice to simulate the physiological environment, but its composition might have been different from that in practical environments. Exposure should differ depending on the site of anastomosis, including the stomach, small intestine, and large intestine. Furthermore, the results *in vitro* could not accurately reflect the results *in vivo*. We performed an *in vivo* experiment, but its safety could not be completely evaluated by using only three porcine intestinal anastomoses as the incidence of anastomotic leakage was low. Long-term safety could not be also evaluated by this short-term observation before the complete degradation of the Mg alloy staples. Therefore, as the *in vivo* study was preliminary, further studies are needed to examine *in vivo* long-term outcomes.

Mg alloy has been reported to promote regeneration or suppress inflammation^21,35^. Therefore, in our next study, we would like to examine the effect of our original Mg alloy on the anastomotic tissue.

In conclusion, these *in vitro* and *in vivo* preliminary experimental results suggest that Mg alloy staples can meet the requirements of intestinal anastomosis. Therefore, our proposed Mg alloy staple is a promising alternative for Ti alloy staples. Based on our findings, we assert that our Mg alloy staple can overcome the disadvantages presented above and is a safe and feasible biodegradable staple.

## Methods

### Material preparation and design of staples

The proposed Mg alloy staple employs the Mg alloy (Mg 2 wt%, Nd 1 wt% Y, FAsorbMg™) fabricated by Fuji Light Metal Co., Japan., as base material. This alloy has adequate mechanical properties for use as staple due to the addition of Nd and Y, with a yield strength of 260 ± 0 MPa, ultimate tensile strength of 290 ± 1 MPa, and elongation of 22 ± 1%. The alloy billet was extruded into a wire rod 2.0 mm in diameter at 723 K with an extrusion ratio of 28:1, and was annealed at 673 K for 30 min in the atmosphere. The wire rod was then repeatedly cold drawn and annealed until its diameter decreased to 0.25 mm. All annealing was carried out at 673 K for 30 min in the atmosphere. The staples used in this experiment were fabricated by press forming the fine wire, and were designed with corners of more than 0.4 mm in radial curvature to reduce the concentration of stress introduced by stapling and anastomosing, as shown in Supplementary Fig. S1. The diameter, width, height, weight, and surface area of the staple were 0.25 mm, 3 mm, 2.5 mm, 0.6 mg, and 0.06378 cm^2^, respectively.

### Computer simulation

FEA using a simulation code of MSC MARC2017 (MSC Software Co., Los Angeles, California, USA) was carried out to simulate the theoretical deformation behaviors of the Mg alloy staples when stapling and anastomosing for both the conventional U–shaped design in clinical use as well as the proposed design. The respective initial two-dimensional (2D) FEA models are shown in Supplementary Fig. S2. The first model simulated stapling deformation from a U-shape to a B-shape by virtually punching the head of the staple at 1 mm/s, and the distribution of the equivalent plastic strain and stress were determined (Supplementary Fig. S2a). The second model simulated the deformation of the B-shaped staple anastomosing the intestine by pulling its legs horizontally by less than 0.1 mm. The simulation thus determined the distribution of equivalent elastic strain and stress as well as plastic strain (Supplementary Fig. S2b). They were defined as having an elastic–plastic body with a Young’s modulus of 45 GPa, yield stress of σ_0_ = 260 MPa, Poisson’s ratio of ν = 0.3, and coefficient of friction of μ = 0.3.

### *In vitro* experiments

#### Immersion test

Preparation of Extract: To simulate the biodegradable behavior of staples in a physiological environment, Mg alloy staples according to the new design fired with ENDOPATH Stapler Echelon Flex 45 (Ethicon Endo–Surgery, Cincinnati, Ohio, USA) were immersed in the artificial intestinal juice at a ratio of 0.006 g weight of the staple per 1 mL in a humidified atmosphere with 5% CO_2_ at 37 °C. The composition of the juice accorded to the second fluid for the disintegration test in the 17^th^ edition of the Japanese Pharmacopoeia^36^: 250 mL of 0.2 mol/L potassium dihydrogen phosphate, 118 mL of 0.2 mol/L sodium hydroxide, and 632 ml of water (pH 6.8). The solution was replaced twice a week.

Evaluation of *in vitro* degradation behavior: The gross appearance of the Mg alloy staples was observed after one, four, eight, and 12 weeks of immersion. Moreover, the concentrations of the composition of the alloy (Mg, Y, Nd) in the intestinal juice were calculated by inductively coupled plasma mass spectroscopy (ICP–MS; NexION 300D, Perkin Elmer, Waltham, Massachusetts, USA).

Calculation of *in vitro* changes in mechanical force: Tensile tests were conducted to calculate the mechanical force of the immersed Mg alloy staples in the first, fourth, eighth, and 12^th^ weeks of immersion^37^. The immersed staples, which involved passing fluorocarbon wires through each loop of the closed B-shape, were strained by a universal testing machine (EZ-test, Shimadzu, Kyoto, Japan) at a strain rate of 6 mm/min until fracture.

#### Cytotoxicity test

Cytotoxicity was calculated using an extraction method (colony – forming assay) according to ISO 10993–5:2009^34^. The Mg alloy staples were immersed into Eagle’s minimum essential medium supplemented with a non-essential amino acid solution, 1 mmol/L sodium pyruvate, 50 mg/L kanamycin, and 5 vol% fetal bovine serum (M05 medium), as a ratio of a surface area of 3 cm^2^ of the alloy surface (approximately 50 staples) to 1 mL of the medium. Following incubation in a humidified 5% CO_2_ atmosphere at 37 °C for 24 h, the resultant extract was centrifuged at 3500 rpm for 5 min to collect the resulting supernatant, which was analyzed by ICP–MS to measure Mg concentration. This extract was then diluted in the M05 medium to seven levels of test solutions (0, 3.13, 6.25, 12.5, 25, 50, and 100% of the extract).

Chinese hamster lung fibroblasts (V79 cells), obtained from the JCRB Cell Bank (National Institute of Biomedical Innovation, Osaka, Japan), were distributed in 24 well plates at 50 cells/well. After 6 h of culture in the humidified 5% CO_2_ atmosphere at 37 °C, the medium was replaced by each test solution. These cells were fixed at a 10% neutral buffered formalin solution and stained with a 0.1% methylene blue solution on the sixth day of incubation. The number of colonies with 50 or more cells was recorded. The rate of colony formation was expressed as the ratio of the number of colonies in each solution to that in pure medium control.

### *In vivo* experiment

Three female adult pigs with a mean body weight of 35.7 ± 0.9 kg were used for the experiment. Under general anesthesia with isoflurane at the end-tidal concentration of approximately 1.5%–3%, we performed stapled functional end–to–end anastomosis using Mg alloy staples and clinically available Ti alloy staples (Ti6Al4V Alloy, Ethicon Endo–Surgery, Inc., Cincinnati, Ohio, USA), respectively, as shown in Supplementary Fig. S3. Both types of staples were loaded into the Echelon Flex Powered ENDOPATH Stapler (Ethicon Endo–Surgery, Cincinnati, Ohio, USA), and all operative procedures were carried out under sterile conditions. These pigs were used in the first, second, and fourth postoperative weeks to evaluate the incidence of anastomotic leakage, hematoma, and intra-abdominal adhesion. In addition to this macroscopic observation, tissue samples of small intestines anastomosed by two types of staples were histopathologically evaluated by pathologists blinded to the material of the staples. Tissue samples were formalin fixed, embedded in resin, and cut into 30-µm-thick slices. These consecutive resin embedded sections were stained with hematoxylin–eosin.

This study was approved by the IVTeC Animal Experiment Committee (Approval ID: IVT15–17). All experiments were performed in accordance with relevant guidelines and regulations.

## Supplementary information

supplementary information

## Data Availability

The datasets generated and/or analyzed during the current study are available from the corresponding author on reasonable request.
