# Supplementary material for: Biodegradable Surgical Staple Composed of Magnesium Alloy
Source: Sci Rep. 2019 Oct 11;9:14671. doi: 10.1038/s41598-019-51123-x (PMC6789124; doi:10.1038/s41598-019-51123-x)
Supplement: Supplementary file 1 — supplementary information [file 41598_2019_51123_MOESM1_ESM.docx]

**Biodegradable Surgical Staple Composed of Magnesium Alloy**

Hizuru Amano^a^, Kotaro Hanada^b^, Akinari Hinoki^c^, Takahisa Tainaka^c^, Chiyoe Shirota^c^, Wataru Sumida^c^, Kazuki Yokota^c^, Naruhiko Murase^c^, Kazuo Oshima^c^, Kosuke Chiba^c^, Yujiro Tanaka^c^, Hiroo Uchida^c, *^

^a^ Department of Pediatric Surgery, Graduate School of Medicine, The University of Tokyo, Tokyo 113-8655, Japan.

^b^ Advanced Manufacturing Research Institute, National Institute of Advanced Industrial Science and Technology (AIST), Ibaraki 305-8564, Japan.

^c^ Department of Pediatric Surgery, Nagoya University Graduate School of Medicine, Nagoya 466-8550, Japan.

*Corresponding author.

Hiroo Uchida M.D., Ph.D.

Professor and Chairman

Department of Pediatric Surgery

Nagoya University Graduate School of Medicine

65 Tsurumai, Showa, Nagoya 466-8550, Japan

Email: hiro2013@med.nagoya-u.ac.jp

Tel.: +81-52-744-2959


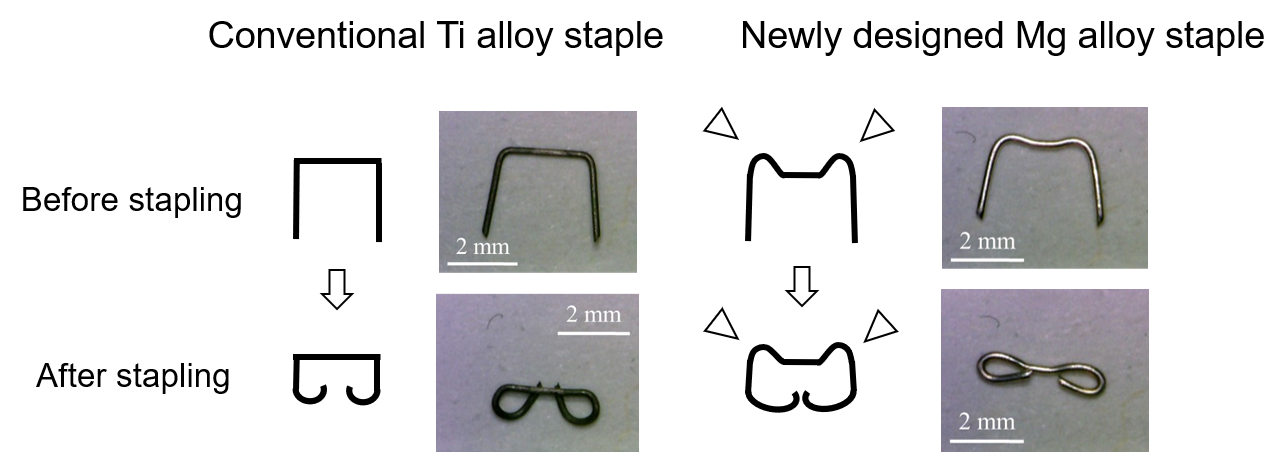


**Supplementary Fig. S1** Configurations of a conventional Ti alloy staple clinically utilized and our newly designed Mg alloy staple. The corners of the developed staple form a curved shape with no bending points (arrowheads), whereas the conventional staple is U–shaped.


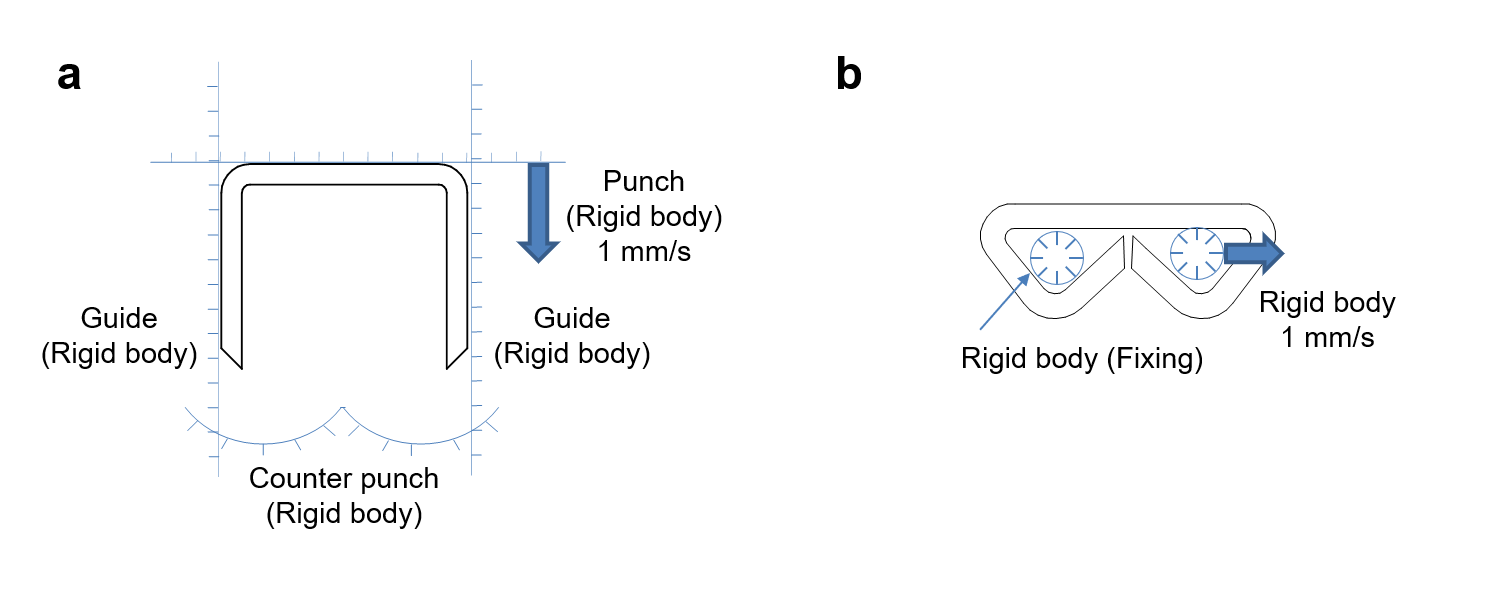


**Supplementary Fig. S2** Finite element analysis models of the deformation behavior of the designed staples. Schematic of (a) deformation to a B-shape and (b) B-shape retention.


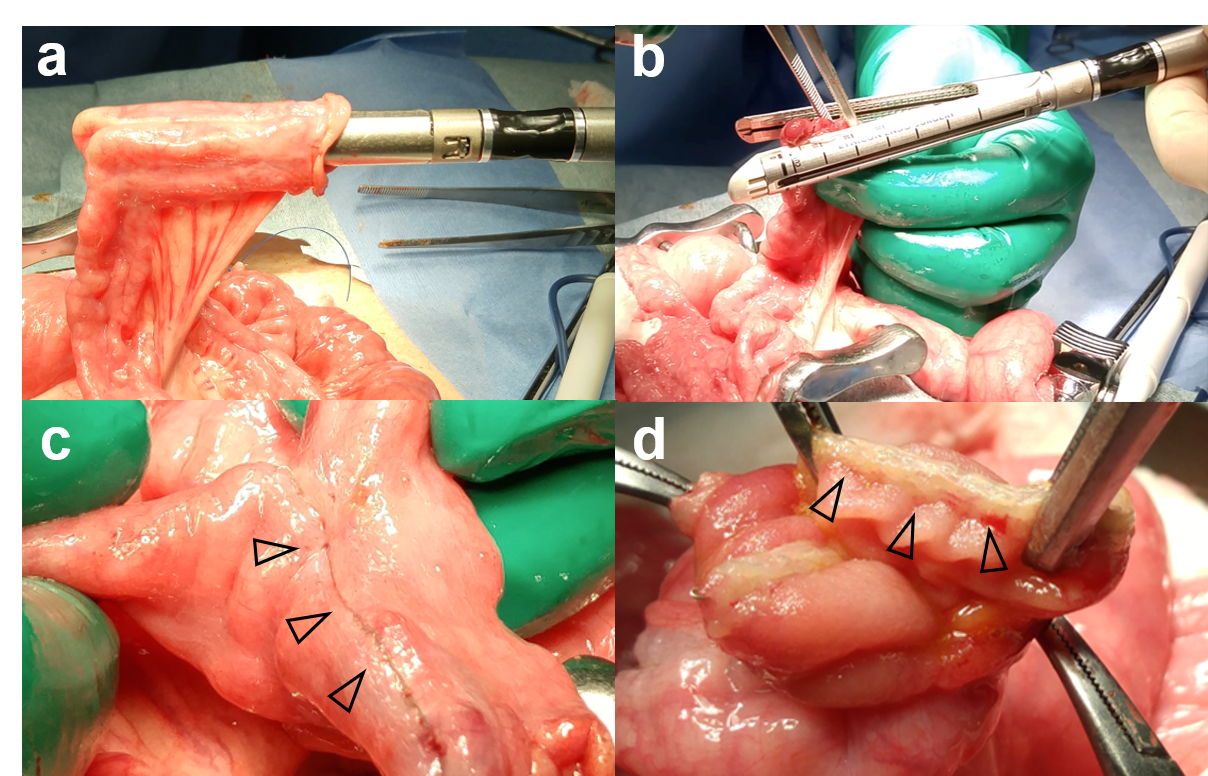


**Supplementary Fig. S3** Surgical procedure of stapled side-to-side functional end-to-end anastomosis (a, b) and staple lines after intestinal anastomosis using the developed Mg alloy staple (c, d).

(a) Proximal and distal intestinal limbs are sutured together, and the stapler is inserted into the intestinal limbs. A side-to-side anastomosis is made in both limbs at the anti-mesenteric border. (b) The stapler is fired again across the joined intestinal limbs to close the enterotomies. (c) First staple line and (d) second staple line using the developed Mg alloy staple.
